# Dispositional individual differences in cognitive effort investment: establishing the core construct

**DOI:** 10.1186/s40359-021-00512-x

**Published:** 2021-01-22

**Authors:** Corinna Kührt, Sebastian Pannasch, Stefan J. Kiebel, Alexander Strobel

**Affiliations:** Faculty of Psychology, Technische Universität Dresden, Zellescher Weg 17, 01062 Dresden, Germany

**Keywords:** Effort discounting, Demand avoidance, Need for cognition, Self-control, Motivation, Effort investment, Individual differences

## Abstract

**Background:**

Individuals tend to avoid effortful tasks, regardless of whether they are physical or mental in nature. Recent experimental evidence is suggestive of individual differences in the dispositional willingness to invest cognitive effort in goal-directed behavior. The traits need for cognition (NFC) and self-control are related to behavioral measures of cognitive effort discounting and demand avoidance, respectively. Given that these traits are only moderately related, the question arises whether they reflect a common core factor underlying cognitive effort investment. If so, the common core of both traits might be related to behavioral measures of effort discounting in a more systematic fashion. To address this question, we aimed at specifying a core construct of cognitive effort investment that reflects dispositional differences in the willingness and tendency to exert effortful control.

**Methods:**

We conducted two studies (*N* = 613 and *N* = 244) with questionnaires related to cognitive motivation and effort investment including assessment of NFC, intellect, self-control and effortful control. We first calculated Pearson correlations followed by two mediation models regarding intellect and its separate aspects, *seek* and *conquer*, as mediators. Next, we performed a confirmatory factor analysis of a hierarchical model of cognitive effort investment as second-order latent variable. First-order latent variables were cognitive motivation reflecting NFC and intellect, and effortful self-control reflecting self-control and effortful control. Finally, we calculated Pearson correlations between factor scores of the latent variables and general self-efficacy as well as traits of the Five Factor Model of Personality for validation purposes.

**Results:**

Our findings support the hypothesized correlations between the assessed traits, where the relationship of NFC and self-control is specifically mediated via goal-directedness. We established and replicated a hierarchical factor model of cognitive motivation and effortful self-control that explains the shared variance of the first-order factors by a second-order factor of cognitive effort investment.

**Conclusions:**

Taken together, our results integrate disparate literatures on cognitive motivation and self-control and provide a basis for further experimental research on the role of dispositional individual differences in goal-directed behavior and cost–benefit-models.

## Background

People tend to avoid or at least minimize exertion of not only physical [1, 2], but also cognitive effort [3, 4]. The experimental series of Kool et al. [5] demonstrates a general tendency of individuals to avoid cognitive demand across different choice settings and demand manipulations. As monetary reward diminished this tendency, individuals are likely to consider mental effort costly. This conclusion is important for research in motivation and cognitive control assuming that decision-making relies on cost–benefit analyses [6–8]. As the net value of a specific benefit is the result of both its actual and perceived effort to attain the benefit, individuals typically favor less cognitively demanding tasks given the same reward. This is commonly labeled as effort discounting [9–11]. Indeed, two seminal findings indicate that individuals do not discount effort to the same extent. First, Westbrook et al. [11] obtained a positive correlation between Need for Cognition (NFC) and the subjective value of performing tasks of increasing difficulty. Individuals high in NFC showed less effort discounting in a so-called cognitive effort discounting paradigm. Second, Kool et al. [12] showed that when having to choose between two visual patterns that were associated with tasks of differing demand, individuals generally tended to choose the low demand option in this so-called demand selection task. However, individuals high in self-control showed less demand avoidance.

The observed associations of traits with effort discounting behavior raise the following question: are these associations specific for these traits or do they reflect a common core of NFC and self-control, in which both traits involve cognitive effort investment? We outline this question in the following.

NFC describes “[…] an individual’s tendency to engage in and enjoy effortful cognitive endeavors.” [13 p. 306]. NFC correlates positively with dimensions associated with dispositional approach-behavior, activity, and goal orientation (i.e., persistence, competence and achievement striving) [14] as well as achievement motivation [15]. On a psychophysiological basis, Mussel et al. [16] investigated theta oscillations in the electroencephalogram, as they are often used as an indicator of cognitive effort [17]. Individuals high in NFC dynamically recruited cognitive resources in line with task demands, i.e., spent less cognitive effort in easier than in harder tasks. The resulting effect on performance was that individuals high in NFC performed better in the harder condition compared to individuals with lower NFC. Regarding the effort discounting phenomenon, the estimation of costs might contrast cognitive effort with cognitive boredom. Individuals high in NFC might integrate the additional motivational attraction into the cost–benefit-analysis.

Self-Control refers to the capacity of adapting one’s immediate state to achieve higher-order goals [18]. It correlates positively with self-esteem and academic success in terms of grades and negatively with the presence of dysfunctional and impulsive behavior like problematic drinking and eating patterns [18]. The strength model of self-control [19] assumes self-control as a limited, depletive resource (ego depletion effect). A meta-analysis [20] on this effect reveals evidence in favor but also against this view. On the one hand, ego-depletion influences effort and subjective task difficulty. On the other hand, motivational incentives or practicing self-control reduces ego-depletion, whereas the anticipation of an upcoming self-control task strengthens it. Hence, Inzlicht et al. [21] understand ego-depletion as a motivated adaptation to task preferences, as people generally tend to balance their motivation between want-to vs. have-to goals. As to self-control and effort investment, a study of Lindner et al. [22] demonstrated that individuals high in self-control spent consistently more effort during an exam than individuals low in self-control.

Taken together, NFC and self-control seem to represent quite distinct constructs and only a moderate correlation exists between the two traits [23]. As both traits affect effort investment, they may have a common motivational core related to the willingness to invest mental effort. Both traits show associations with conscientiousness [14, 18], goal-directedness [14, 19] and achievement motivation [15, 20], as well as a positive effect on task and school performance [16, 18, 23]. In summary, both constructs seem to share the aspect of goal orientation that the intellect framework [24] contains as described below.

The intellect framework structures individual differences related to intellectual achievement [24]. It consists of two dimensions: processes (*seek* and *conquer*) and operations (*think*, *learn* and *create*). The process *seek* refers to the general openness to approach intellectually demanding situations where one can *think* about something or can *learn* or *create* something new. The process *conquer* captures motivational processes in such situations, i.e., effort, diligence and persistence in thinking, learning or creating something. Experimental findings [25] support the assumption that intellect covers both *seek* and *conquer* processes. Smillie et al. found a positive relation of intellect and task performance variables and emphasize the utility of high intellect for learning and performance. Within the intellect framework, Mussel relates NFC to the process *seek* and the operation *think* based on theoretical considerations and supporting empirical evidence [24]. However, given the aforementioned relationship of NFC with traits reflecting approach-behavior as well as persistence and goal-directedness [14], we assume that NFC additionally relates to the process *conquer*. As *conquer* can be conceptualized as goal-directedness, it might represent the common aspect shared by NFC and self-control due to the outlined theoretical and empirical indications. To sum up, we formulate or first hypothesis (H1) as follows.

### **H1:**

Intellect and particularly *conquer* mediate the relationship of NFC and self-control.

Given the two central findings of Westbrook et al. [11] and Kool et al. [12], we assume that NFC and self-control might be integrated based on the common aspect of cognitive effort investment, i.e., dispositional individual differences in the willingness and tendency to exert effortful control. An integrative model of cognitive effort investment offers the opportunity (a) to address research questions about individual differences in effort discounting and demand avoidance in a systematic fashion and (b) to relate dispositional cognitive effort investment to behavioral measures of demand avoidance. Establishing such a model via confirmatory factor analysis (CFA) requires at least one further scale highly related to NFC (or more generally cognitive motivation) and to self-control (or more generally the effortful exertion of self-control), as the correlated indicators are essential to estimate the latent factors. In the present paper, we address the question whether we can derive a common core construct that (a) provides a *single* measure of cognitive effort investment to relate to behavioral measures of effort discounting and demand avoidance without having to correct for multiple testing. At the same time, this model should (b) allow for retaining a differentiation into cognitive motivation and self-control aspects of cognitive effort investment for the purpose of more fine-grained analyses.

**Fig. 1:**
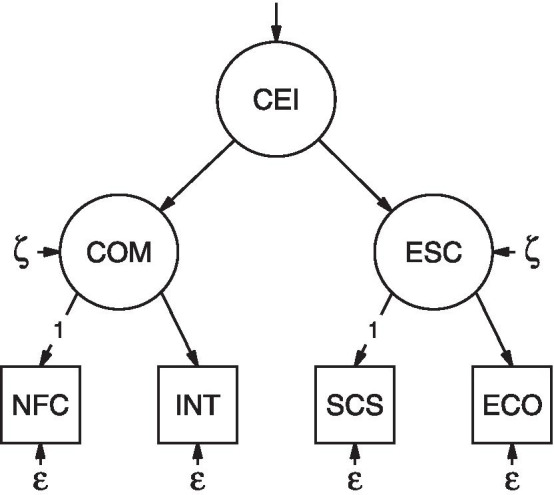
Hypothesized structural model of dispositional cognitive effort investment. *CEI* cognitive effort investment, *COM* cognitive motivation, *ESC *effortful self-control, *NFC* need for cognition, *INT* intellect, *SCS* self-control, *ECO * effortful control, *ξ* residuals of the first-order factors set equal, *ε* error variances of the indicator variables set equal, *1 * loadings of the indicator variables fixed to 1

To this end, we chose (1) intellect as one further trait related to cognitive motivation and (2) effortful control [from the Adult Temperament Questionnaire (ATQ) [26]] as one further trait related to effortful self-control. Intellect is related to NFC as discussed previously. Also, if we could confirm our mediation hypothesis that NFC relates to self-control via intellect, this would strengthen the construct overlap. We selected effortful control because it refers to “[…] the ability to focus attention and shift to desired channels.” [27 p. 3] allowing individuals to act in accordance to their long-term goals even in challenging situations. Just like self-control, effortful control has a negative relation to dysfunctional and impulsive behavior, e.g., problematic buying and eating behavior [28, 29]. On a cognitive level, effortful control is highly related to executive functions, especially to updating and monitoring processes in working memory [30]. Additionally, self-control and effortful control correlate strongly and load on a common self-control factor [31]. In summary, our model of cognitive effort investment contains two main clusters as illustrated in Fig. 1. The first refers to dimensions associated with cognitive motivation (i.e., NFC and intellect). The second is about effortful self-control (i.e., self-control and effortful control). We derive the following hypotheses.

### **H2:**

The intercorrelations of NFC, intellect, self-control and effortful control reflect the hypothesized structure (Fig. 1), i.e. strong correlations between NFC and intellect as well as self-control and effortful control, but medium-sized between the former and the latter traits.

### **H3:**

A pragmatic model of cognitive effort investment as illustrated in Fig. 1 integrates the shared variances of these variables.

We measured general self-efficacy and traits of the Five Factor Model of Personality (FFM) in order to validate the predictive value of our model. General self-efficacy belongs to the general perception of one’s ability to perform new or challenging tasks including coping with daily troubles [32]. Highly self-efficacious individuals are optimistic and self-confident about task performance and outcomes, aim higher, go for more challenging tasks, invest more effort, persist longer, keep their goals and overcome setbacks faster [32–34]. Self-efficacy seems a promising trait in the context of effort investment. We expected a positive relation between general self-efficacy and cognitive effort investment in general as well as both cognitive motivation and effortful self-control in particular.

We included general dimensions of personality into our validating analysis to put cognitive effort investment into a more global context. Personality traits of the FFM provide an appropriate description of higher-order traits including neuroticism, extraversion, openness, agreeableness and conscientiousness [35]. These traits show substantial correlations with the traits we focused on in the present research. Conscientiousness correlates moderately with NFC [14] and intellect [24] and strongly with self-control [18] and effortful control [27]. Accordingly, we assumed conscientiousness to be more closely related to the first-order latent variable of effortful self-control. Openness correlates moderately with NFC [14, 36] and highly with intellect [24]. Especially the facet openness to ideas connects closely to both concepts. According to DeYoung et al. [37], openness and intellect represent aspects of the same fundamental construct. On this basis, the authors define openness/intellect “[…] as motivated cognitive flexibility, or cognitive exploration […]” [37 p. 850 ff] being associated with general cognitive ability, and specifically with fluid intelligence. This suggests that openness relates closely to the first-order latent variable of cognitive motivation. Neuroticism correlates moderately with NFC [14, 36] as well as strongly negative with self-control [18], and effortful control [27, 30]. This indicates a stronger relation to the latent first-order variable of effortful self-control. The association of extraversion with NFC is at best moderate [36], as it is with effortful control [27]. Thus, we expected extraversion to be at least slightly related to the overall latent variable of cognitive effort investment. Agreeableness displays a moderate correlation with self-control [18] and effortful control [27], indicating a small relation to the first-order latent variable of effortful self-control. H4 summarizes these considerations as follows.

### **H4:**

Relevant traits, i.e. general self-efficacy and personality traits of the FFM, intercorrelate with the construct of cognitive effort investment.

In light of the research replication crisis [38], we deem exact replications an important aspect of a study [39–41]. In order to demonstrate the reproducibility of our findings, we tested all hypotheses in two independent samples, i.e., Study 1 and 2.

The main aim of the present paper was to establish a reliable and pragmatic model of cognitive effort investment for further studies, based on the view of cognitive effort investment as self-reported dispositional differences in the willingness and tendency to exert effortful control. To this end, this paper addresses the following questions: What have NFC and self-control in common (H1)? Can we integrate effort related traits into one general measure of cognitive effort investment (H2, H3)? Is this general measure associated with other effort-related traits (H4)? Are the findings reproducible and therefore reliable (replication of H1–H4)?

## Study 1

### Methods

We report how we determined our sample size, all data exclusions (if any), all manipulations, and all measures in the study [cf. 42]. The dataset and all analysis scripts for Study 1 are available on OSF website [43].

#### Participants

In total, 613 participants (70% female, 85% university entrance diploma, 31% students; age M ± SD: 29.1 ± 10.8 years (see Additional file 1: Supplement A for socio-demographic details) completed an online assessment. All participants gave informed consent in accordance with the Declaration of Helsinki upon entering the online questionnaire. The ethics board of the Technische Universität Dresden (TUD) approved the study protocol, *reference number:* EK 3012016.

#### Procedure

We recruited participants via social networks, websites and advertisements at the TUD and other German universities. There existed no ad hoc inclusion nor exclusion criteria. We aimed for a sample size of *N* = 782 to detect small correlations (*r* = 0.10, α = 0.05, β = 0.80). At a total of *N* = 923 entries we stopped recruiting due to time constraints, but had to register *n* = 310 incomplete questionnaires, leaving us with a final sample size of *N* = 613 that was used for all analyses. This still allowed us to detect correlations of *r* = 0.11 (α = 0.05, β = 0.80). The online assessment lasted approximately 25 min. Participants optionally registered for a lottery (possible winnings: 1 × 100 Euro, 2 × 50 Euro, 4 × 25 Euro).

#### Materials

We included the following questionnaires.

*Sociodemography.* Multiple-choice questions assessed age, sex, graduation, qualification and field of study.

*NFC*. The 16-item short version of the German NFC Scale [15] assessed NFC. Responses to each item (e.g., “I really enjoy a task that involves coming up with new solutions to problems.” [44]) were recorded on a 7-point Likert scale ranging from − 3 (disagree strongly) to + 3 (agree strongly). The scale shows comparably high internal consistency with Cronbach’s *α* > 0.80 [14, 15], and a retest reliability of *r*_*tt*_ = 0.83 across 8 to 18 weeks [45].

*Intellect*. To measure intellect, we employed the 24-item Intellect Scale by Mussel [24]. It assesses two intellectual processes (*seek* and *conquer*) and three intellectual operations (think, learn, and create). Items (e.g., "I enjoy solving complex problems" for the *seek/think* facet or "When I’m developing something new, I can’t rest until it’s completed" for the facet of *create/conquer*) are rated on a 7-point Likert scale ranging from − 3 (disagree strongly) to + 3 (agree strongly). Internal consistency is high (Cronbach's *α* = 0.94 for the total Intellect score and ≥ 0.86 for the six facets [24]), and 1-year retest reliability is acceptable (*r*_*tt*_ = 0.73 for the total Intellect score and ≥ 0.58 for the six facets) (Mussel P, personal communication, June 24, 2020).

*Self-control.* Self-control was measured using the short form of the German Self-Control Scale [46] that comprises 13 items (e.g., “I am able to work effectively toward long-term goals”) with a 5-point Likert scale ranging from − 2 (disagree strongly) to + 2 (agree strongly). The scale shows comparably high reliability (Cronbach’s *α* ~ 0.80, 7-week retest reliability *r*_*tt*_ = 0.82 [23]).

*Effortful Control.* The respective scale of the German ATQ [27] assessed effortful control. It comprises 19 items on executive control in everyday life. Responses to items (e.g., “Even when I feel energized, I can usually sit still without much trouble if it’s necessary”) are given on a 7-point Likert scale from − 3 (disagree strongly) to + 3 (agree strongly). Internal consistency of the scale is acceptable (Cronbach’s *α* = 0.74 [27]), and 5-week retest reliability is good with *r*_*tt*_ = 0.80 [47].

We provide details on construct validity of these questionnaires in supplemental materials (Additional file 1: Supplement B). In addition, we used the following scales for validation purposes.

*General self-efficacy.* We applied the German version of the General Self-Efficacy Scale [48] to record the general sense of perceived competence to cope with manifold stressful situations. It contains 11 items (e.g., “I can usually handle whatever comes my way.”) with response option on a 4-point scale ranging from 1 (not at all true) to 4 (exactly true). Internal consistency ranges from good to excellent (Cronbach’s alpha = 0.82–0.93), whereas 2-year retest reliability is poor (*r*_*tt*_ = 0.47 for men and *r*_*tt*_ = 0.63 for women) [48].

*Higher-order traits.* The Big Five Inventory Short Form (BFI-K) [49] assessed neuroticism, extraversion, openness to experience, agreeableness and conscientiousness. This economic operationalization of the FFM consists of 21 items (e.g. “I see myself as someone who is curious about many different things” for the factor Openness) and contains a 5-point Likert scale ranging from 1 (disagree strongly) to 5 (agree strongly). Internal consistencies range from acceptable to good (Cronbach’s *α* = 0.64—0.86); retest reliability is comparably good *r*_*tt*_ = 0.84 [49].

#### Statistical analyses

All analyses were performed using RStudio [50] with R 3.6.1 [51] and packages *psych* (version 1.8.12) [52] and *lavaan* (version 0.6.5) [53] (see Additional file 1: Supplement C for additional packages used). As some of the trait variables deviated from univariate normality (Shapiro–Wilk tests, *p* ≥ 0.20), all variables were normalized using Blom’s formula [(*r − *3/8)/(*n* + 1/4), with *r* being the rank of observations and *n* the sample size] [54] and standardized. The normalized variables did not deviate from univariate normality (Shapiro–Wilk tests, *p* ≥ 0.20), except for the dimensions of the FFM and general self-efficacy (Shapiro–Wilk tests, *p* ≤ 0.05) (see Additional file 1: Supplement D).

First, to test H1, we tested mediation models based on the manifest variables. Therefore, we estimated a model with the intellect total score as mediator. To assess whether the two intellect aspects *seek* and *conquer* show different mediator effects, we estimated a model with these two variables as mediators, with their residuals allowed to correlate. Please note that the assignment of NFC as independent variable and self-control as dependent variable does not reflect theoretical considerations and, in fact, is interchangeable. We were simply interested in whether the relation between NFC and self-control is due to their relation to intellect. Mediation models were estimated using maximum likelihood estimation with robust (Huber-White) standard errors.

Second, to test H2, we calculated correlations as Pearson correlations with p-values corrected for multiple testing using Holm’s method [55].

Third, to test H3, we performed a CFA with the NFC score and the intellect total score as indicator variables of the latent variable cognitive motivation and the self-control and effortful control scores as indicator variables for the latent variable effortful self-control. From these latent variables, we derived a higher-order latent variable cognitive effort investment. As to indicator variables, we fixed loadings of the first indicator per latent variable (i.e., NFC and self-control) to one and set error variances to equal. We fixed the loadings of first-order factors on the second-order factor cognitive effort investment to one, and set the residuals of first-order factors to equal. Note that we determined this model specification during exploratory data analysis. Therefore, we replicated the analysis using the same model in Study 2. Model parameters were estimated using maximum likelihood estimation with robust (Huber-White) standard errors. Model fit was evaluated via the scaled estimates of the scaled comparative fit index (CFI), scaled root mean square error of approximation (RMSEA), and standardized root mean square residual (SRMR), with values of CFI ≥ 0.95, RMSEA ≤ 0.06, and SRMR ≤ 0.08 indicating good model fit [56]. Reliability of the factors was determined as McDonald’s ω. We tested this model against a non-hierarchical model with two correlated factors using scaled χ^2^ difference tests.

Fourth, to test H4, we calculated correlations as Pearson correlations between the factor scores of latent variables (i.e., cognitive motivation, effortful self-control and cognitive effort investment) and general self-efficacy as well as dimensions of the FFM (i.e., neuroticism, extraversion, openness, agreeableness and conscientiousness). Again, we corrected for multiple testing using Holm’s method.

### Results

*Correlation analysis.* Table 1 shows the intercorrelations and internal consistencies of the trait variables. As expected, NFC and intellect were highly correlated, *r* = 0.70, *p* < 0.001, as were self-control and effortful control, *r* = 0.74, *p* < 0.001, while the correlations between the former two scales and the latter measures were only moderate, 0.28 < *r* < 0.37, *p* < 0.001.

**Table 1:** Intercorrelations and internal consistencies of all variables (Study 1)

Variable		1	2	3	4	5	6
1	NFC	*.87*					
2	Intellect	.70	*.96*				
3	Seek^a^	.71	.94	*.93*			
4	Conquer^a^	.61	.95	.78	*.93*		
5	Self-control	.28	.31	.26	.33	*.83*	
6	Effortful control	.34	.37	.35	.35	.74	*.83*

Coefficients are Pearson correlations (all *p* < .001); values in the diagonal give the internal consistencies (Cronbach’s α)

^a^Correlations with *seek* and *conquer* are not part-whole-corrected, part-whole corrected correlations equal the bivariate correlation between *seek* and *conquer*

**Fig. 2:**
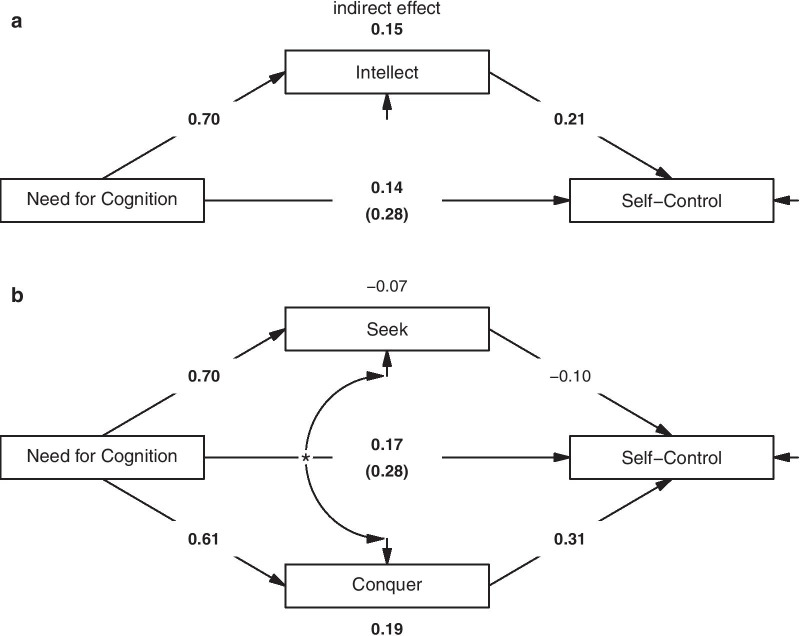
Mediation models (Study 1). **a** Mediator effect of the intellect total score. **b** Mediator effects of the intellect aspects—*seek* and *conquer*. Unstandardized coefficients are given. Significant paths are bold-faced. Residuals are not shown for reasons of simplicity. Note that the residuals of *seek* and *conquer* are significantly correlated (*)

*Mediation models.* Figure 2 depicts the mediation models based on manifest variables. Trait intellect partially mediated the relationship between NFC and self-control, indirect effect = 0.15, 95% CI [0.07, 0.23], *p* < 0.001; direct effect = 0.14, 95% CI [0.02, 0.25], *p* = 0.017. When using the intellect subscales *seek* and *conquer* as separate mediators, the relationship between NFC and self-control was specifically mediated via the *conquer* aspect of intellect, indirect effect = 0.19, 95% CI [0.10, 0.27], *p* < 0.001, but not via its *seek* aspect, indirect effect = − 0.07, 95% CI [− 0.18, 0.04], *p* = 0.232, direct effect = 0.17, 95% CI [0.05, 0.28], *p* = 0.004.

**Fig. 3:**
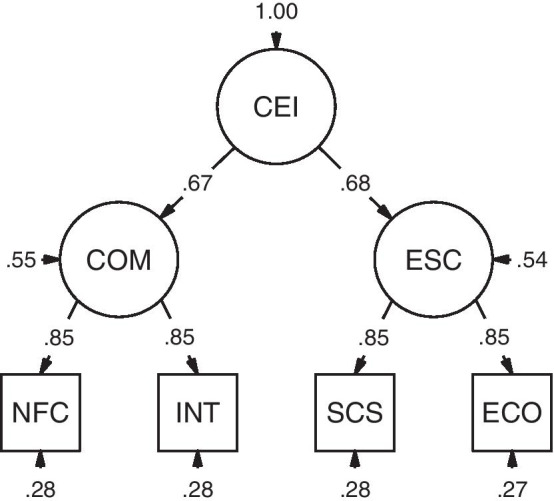
Structural model of dispositional cognitive effort investment in Study 1 (completely standardized solution). *CEI* cognitive effort investment, *COM* cognitive motivation, *ESC* effortful self-control, *NFC* need for cognition, *INT* intellect, *SCS* self-control, *ECO* effortful control

*CFA.* We conducted a CFA for the hierarchical model (Fig. 3) that showed a good fit, *χ*^2^ = 8.94, *df* = 4, *p* = 0.063, CFI = 0.99, RMSEA = 0.04 with 90% CI [0.00, 0.08], SRMR = 0.02. Additionally, we estimated the parameters of a non-hierarchical model with two correlated first order factors. This model showed a very similar fit, *χ*^2^ = 9.41, *df* = 5, *p* = 0.094, CFI = 0.99, RMSEA = 0.04 with 90% CI [0.00, 0.07], SRMR = 0.02, that was not different from that of the hierarchical model, *χ*^2^_diff_ = 0.07, *df*_diff_ = 1, *p* = 0.794.

*Validation analysis.* Table 2 depicts intercorrelations of the factor scores derived from the hierarchical model with general self-efficacy and dimensions of the FFM. The cognitive effort investment factor score substantially correlated with general self-efficacy, *r* = 0.57, *p* < 0.001. Here, the first-order factors showed equal-sized associations with general self-efficacy, both *r* = 0.50, *p* < 0.001. Second-order cognitive effort investment was substantially related with conscientiousness, *r* = 0.65, *p* < 0.001, mainly due to a strong relation of the latter with first-order effortful self-control, *r* = 0.68, *p* < 0.001. To a lesser extent, it was also associated with openness, *r* = 0.30, *p* < 0.001, mainly due to a correlation of the latter with first-order cognitive motivation, *r* = 0.43, *p* < 0.001. Moreover, cognitive effort investment was negatively correlated with neuroticism, *r* = − 0.38, *p* < 0.001, mainly due to a higher correlation of neuroticism with first-order effortful self-control, *r* = − 0.43, *p* < 0.001.

**Table 2:** Intercorrelations and internal consistencies of factor scores, general self-efficacy and higher-order variables (Study 1)

Variable		1	2	3	4	5	6	7	8	9
1	Cognitive Effort Investment	*.88*								
2	Cognitive Motivation	.87***	*.84*							
3	Effortful Self-Control	.88***	.52***	*.84*						
4	General Self-Efficacy	.57***	.50***	.50***	*.88*					
5	Neuroticism	− .38***	− .23***	− .43***	− .53***	*.82*				
6	Extraversion	.11*	.19***	.01	.39***	− .27***	*.84*			
7	Openness	.30***	.43***	.11*	.16**	.04	.21***	*.76*		
8	Agreeableness	.15**	.08	.18***	.11*	− .15**	.19***	.08	*.64*	
9	Conscientiousness	.65***	.46***	.68***	.45***	− .20***	.16***	.16***	.16**	*.71*

Coefficients are Pearson correlations (***p < .001; **p < .01; *p < .05); values in the diagonal give reliability estimates (McDonald’s ω for entries 1–3 and Cronbach’s α for entries 4–9)

### Discussion

In the following, we first summarize our main findings of Study 1 with respect to our individual hypotheses. The General Discussion will go into more detail on both studies. The results of Study 1 support our hypotheses. First, the relation of NFC and self-control was partially mediated via trait intellect, precisely via its *conquer* aspect but, as hypothesized, not via its *seek* aspect (H1). Second, traits related to effort investment intercorrelated in the expected manner (H2), i.e., strong associations between NFC and intellect, as between self-control and effortful control, but moderate associations between the two former and the two latter scales. Third, we found the proposed structure of the core construct of cognitive effort investment (H3) fitted with our data, i.e. we integrated traits related to effort investment into a hierarchical factor model with the first-order factors cognitive motivation and effortful self-control and the second-order factor cognitive effort investment. In the event of a non-significant difference in fit between a non-hierarchical and the hierarchical model, one typically prefers the simpler model. However, it was exactly our aim to assume a second-order factor and given its good fit with the data, we continued to prefer the hierarchical model, although the hierarchical nature was simply a speculation at this point. Fourth, we found that cognitive effort investment substantially correlated with general self-efficacy as well as conscientiousness, openness and neuroticism of the FFM (H4). The differentiation in first- and second-order factors also enabled us to examine in more detail which aspect of cognitive effort investment correlated more strongly with the respective broad personality trait. This indicates the validity of the model to predict other traits that are relevant in the context of effort investment. One limitation of Study 1 is that we selected some specifications of the hierarchical factor model such as imposing model constraints during data analysis. For a truly CFA, we needed to test the final model in a separate sample, see Study 2.

## Study 2

Study 2 was a direct replication of Study 1. We applied the same measures in an online assessment in order to validate the hierarchical factor model and run exactly the same statistical analyses.

### Methods

We report how we determined our sample size, all data exclusions if any), all manipulations, and all measures in the study [cf. 42]. The dataset and all analysis scripts for Study 2 are accessible at OSF website [43].

#### Participants

244 participants (72% female, 99% university entrance diploma, 86% students, 13% with job; age M ± SD: 23.4 ± 4.3 years (see Additional file 1: Supplement A for socio-demographic details) completed the online assessment used in Study 1 on tablet in an experimental setting. They signed a written informed consent form in accordance with the Declaration of Helsinki. The ethics board of the TUD approved the study protocol, *reference number:* EK 3012016.

#### Procedure

We recruited participants via university websites for online inscription to psychological research and advertisements at Dresden’s universities. Inclusion criteria were an age between 18 and 38 years, speaking German fluently, right-handedness, normal or normal-corrected vision. Exclusion criteria were psychological, psychiatric or neurological dysfunctions, substance misuse, psychotropic medication, and other reasons for non-participation. We aimed at a sample size of *N* = 200 for other analyses reported separately [47]. To detect correlations of *r* = 0.28 as obtained for NFC and self-control in Study 1 an *N* = 97 is appropriate (α = 0.05, β = 0.80). The total sample size of *N* = 244 completed questionnaires is sensitive to correlations of *r* = 0.18 (α = 0.05, β = 0.80) and was used for all analyses. The online assessment used in Study 1 was embedded in a laboratory experiment with two appointments separated by an interval of 5 weeks. At both appointments, participants performed a cognitive task battery (20 min), two versions of a demand selection task (30 min each), responding questionnaires and the online assessment (25 min) presented on tablet. Each appointment lasted approximately 2 h. Participants received 8 euro per hour plus 10 euro extra for attending a second appointment.

#### Materials

We applied exactly the same questionnaires as in Study 1. Note that the sociodemographic questions in Study 2 differed slightly and assessed age, sex, graduation and current job.

#### Statistical analyses

We performed all analysis steps (correlation analysis, mediation analyses, CFA, and validation analysis) as described in Study 1, using the responses of the online assessment during the first appointment only. The normalized variables did not deviate from univariate normality (Shapiro–Wilk tests, *p* > 0.20), except for the dimensions of the FFM (Shapiro–Wilk tests, *p* ≤ 0.20) (see Additional file 1: Supplement E).

### Results

*Correlation analysis*. Table 3 displays intercorrelations and internal consistencies of trait variables. As expected, NFC and intellect were highly correlated, as were self-control and effortful control, both *r* = 0.64, *p* < 0.001, while the correlation between the former two scales and the latter measures were only moderate, 0.26 < *r* < 0.30, *p* < 0.001.

**Table 3:** Intercorrelations and internal consistencies of all variables (Study 2)

Variable		1	2	3	4	5	6
1	NFC	*.86*					
2	Intellect	.64***	*.93*				
3	Seek^a^	.66***	.91***	*.88*			
4	Conquer^a^	.53***	.93***	.71***	*.88*		
5	Self-Control	.30***	.27***	.20**	.29***	*.79*	
6	Effortful Control	.30***	.26***	.22**	.28***	.64***	*.78*

Coefficients are Pearson correlations (***p < .001; **p < .01; *p < .05); values in the diagonal give reliability estimates (Cronbach’s α)

^a^Correlations intellect with *seek* and *conquer* are not part-whole-corrected, part-whole corrected correlations equal the bivariate correlation between *seek* and *conquer*

**Fig. 4:**
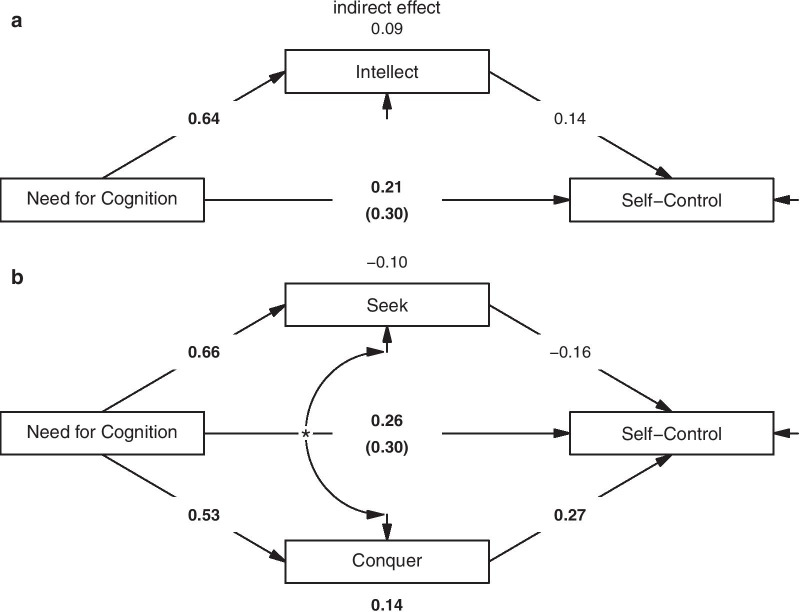
Mediation models (Study 2). **a** Mediator effect of the intellect total score. **b** Mediator effects of the intellect aspects—*seek* and *conquer*. Unstandardized coefficients are given. Significant paths are bold-faced. Residuals are not shown for reasons of simplicity. Note that the residuals of *seek* and *conquer* are significantly correlated (*)

*Mediation models.* Fig. 4 displays the mediation models based on manifest variables. In this sample, the relationship between NFC and self-control was not significantly mediated via intellect, indirect effect = 0.09, 95% CI [− 0.02, 0.20], *p* = 0.122, direct effect = 0.21, 95% CI [0.04, 0.38], *p* = 0.015. However, when including *seek* and *conquer* as separate mediators in the model, the relationship between NFC and self-control was again specifically mediated via the *conquer* aspect of intellect, indirect effect = 0.14, 95% CI [0.06, 0.23], *p* = 0.001, but not via its *seek* aspect, indirect effect = − 0.10, 95% CI [− 0.23, 0.02], *p* = 0.092, direct effect = 0.26, 95% CI [0.09, 0.43], *p* = 0.003.

**Fig. 5:**
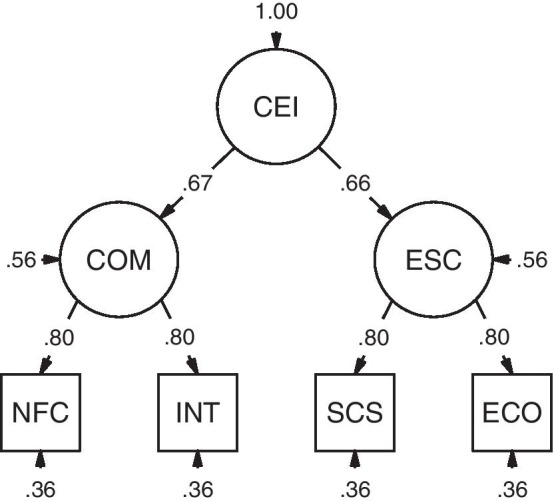
Structural model of dispositional cognitive effort investment in Study 2 (completely standardized solution). *CEI* cognitive effort investment, *COM* cognitive motivation, *ESC* effortful self-control, *NFC* need for cognition, *INT* intellect, *SCS* self-control, *ECO* effortful control

*CFA.* A CFA of the hierarchical model (Fig. 5) yielded an excellent fit, *χ*^2^ = 0.35, *df* = 4, *p* = 0.986, CFI = 1, RMSEA = 0.00 with 90% CI [0.00, 0.00], SRMR = 0.01. Additionally, we fitted a non-hierarchical model, *χ*^2^ = 0.37, *df* = 5, *p* = 0.996, CFI = 1, RMSEA = 0.00 with 90% CI [0.00, 0.00], SRMR = 0.01. This fit did not differ from that of the hierarchical model, *χ*^2^_diff_ < 0.01, *df*_diff_ = 1, *p* = 0.965.

*Validation analysis.* Table 4 shows intercorrelations of factor scores, general self-efficacy and traits of the FFM. The cognitive effort investment factor score significantly correlated with general self-efficacy, *r* = 0.44, *p* < 0.001. In detail, the first-order factors showed equal-sized associations with the latter, 0.37 < *r* < 0.40, *p* < 0.001. Second-order cognitive effort investment was strongly associated with conscientiousness, *r* = 0.56, *p* < 0.001, mainly due to a substantial relation of the latter with first-order effortful self-control, *r* = 0.67, *p* < 0.001. Cognitive effort investment was not associated with openness, *r* = 0.09, *p* = 1, albeit a correlation of the latter with first-order cognitive motivation existed, *r* = 0.21, *p* = 0.018. Its correlation with neuroticism was medium-sized, *r* = − 0.30, *p* < 0.001, and almost equal-sized with first-order latent variables, − 0.27 < *r* < − 0.25, *p* = 0.001 and *p* = 0.002.

**Table 4:** Intercorrelations and internal consistencies of factor scores, general self-efficacy and higher-order variables (Study 2)

Variable		1	2	3	4	5	6	7	8	9
1	Cognitive effort investment	*.84*								
2	Cognitive motivation	.87***	*.78*							
3	Effortful self-control	.88***	.53***	*.78*						
4	General self-efficacy	.44***	.40***	.37***	*.80*					
5	Neuroticism	− .30***	− .25**	− .27**	− .49***	*.76*				
6	Extraversion	.06	.11	− .01	.22*	− .22**	*.81*			
7	Openness	.09	.21*	− .06	.11	.03	.13	*.70*		
8	Agreeableness	.07	.02	.11	.02	− .14	.25**	.10	*.65*	
9	Conscientiousness	.56***	.31***	.67***	.22*	− .04	.05	− .13	.08	*.69*

Coefficients are Pearson correlations (*** *p* < .001, ** *p* < .01, * *p* < .05); values in the diagonal give reliability estimates (McDonald’s ω for entries 1–3 and Cronbach’s α for entries 4–9)

### Discussion

In the following, we first summarize our main findings of Study 2 with respect to our individual hypotheses. The General Discussion will go into more detail on both studies. The findings of Study 2 mainly support our hypotheses and replicate the results of Study 1. First, although trait intellect did not mediate the relation of NFC and self-control in this sample, we again found that the *conquer* aspect did and the *seek* aspect did not meditate this relation (H1). Second, traits related to effort investment intercorrelated in line with our H2, albeit lower in size compared to correlations found in Study 1. Thus, the hypothesized mediating effect by *conquer* was present indicating that NFC and self-control share an aspect that can be conceptualized as goal-directedness. Third, we succeeded in replicating the speculative, hierarchical nature of the factor model of cognitive effort investment (H3). Again, its fit was not different from that of a simpler nonhierarchical model. Nonetheless, the usefulness of an integrative variable is reasonably appropriate for preferring the hierarchical model. Fourth, effort-related traits correlated with relevant latent variables of the model (H4), while the obtained correlations were smaller as compared to Study 1. The main reason for differences in the results compared to Study 1 might be due to the homogeneous sample.

## General discussion

Results of Study 1 and Study 2 support our original hypotheses. As to H1, we found that intellect partially mediates the relationship between NFC and self-control in Study 1, but not in Study 2. The non-significant effect might be due to the smaller and more homogeneous sample of Study 2. At the process level, we found *conquer* mediating the relationship between NFC and self-control in both studies. These results suggest that goal-directedness is the aspect that both NFC and self-control have in common. This is relevant in the context of effort discounting and provides new insights into the conceptual scope of NFC. In contrast to the assumptions of the intellect framework [24], we interpret our findings such that NFC is not restricted to the *seek* aspect of intellect. Instead, it also overlaps with the process of *conquer*. This finding should be considered in the conceptualization of the intellect framework in order to secure the validity of the intellect scale.

Regarding H2, intercorrelations in both studies revealed the hypothesized and robust associations between effort-related traits pointing to a core construct of cognitive effort investment. In detail, we obtained high correlations between NFC and intellect as well as between self-control and effortful control. Associations between the former and the latter two variables were only moderate. In line, Bertrams and Dickhäuser [23] found NFC and self-control moderately related with each other. Moreover, Mussel [24] observed the same strong relationship between NFC and intellect.

Regarding H3, the confirmatory factor analyses showed that the hypothetical structure of cognitive effort investment fitted well with our data and could be replicated. Based on the findings that self-control and NFC play a crucial role in demand avoidance and effort discounting [11, 12], but have been regarded as quite distinct constructs [23], we were able to integrate NFC and self-control into a pragmatic and replicable hierarchical factor model of cognitive effort investment. Comparison against a simpler non-hierarchical model showed that in both samples, such a model showed a nearly indistinguishable fit with the data as the hierarchical model. Although one could argue that in such a case, the simpler model is to be preferred, we regard a hierarchical model as more practical. It was the very aim of the present work to derive an integrative measure of cognitive effort investment in order to relate it to behavioral measures of demand avoidance in further studies. Following up on the results of Westbrook et al. and Kool et al. [11, 12] in one study would require to always correlate both measures—NFC and self-control—with behavioral variables and to correct for multiple testing. This would not be the case, if we have an integrative measure of cognitive effort investment. At the same time, the model still allows discriminating between cognitive motivation and effortful self-control, and thus enables researchers to assess both common and distinct aspects of cognitive effort investment in a parsimonious fashion.

Regarding H4, first- and second-order factors of our model correlated substantially with relevant variables in both studies. In detail, first- and second-order factors revealed substantial correlations with general self-efficacy. These findings are in line with previous studies [32–34] proving the broad character of general self-efficacy and its benefits in highly demanding situations in a motivational, self-controlling as well as goal-directed manner. As to traits of the FFM, cognitive effort investment substantially correlated with conscientiousness, openness (Study 1 only) and neuroticism. In addition, the model provided differentiated information about the association of these traits with first-order factors. As expected, cognitive motivation correlated more strongly with openness, whereas effortful self-control revealed stronger relations to conscientiousness and neuroticism. However, correlations of openness were smaller than expected, and in Study 2 only significant for cognitive motivation. Reported correlations of openness with NFC and intellect [14, 24, 36, 57] contrast with the comparably low correlations in our studies. Recent research of DeYoung et al. [58] on Big Five traits suggest the coexistence of two different aspects within each dimension of the FFM. This finding based on factor analyses of domain’s facets and is backed up by an essential and reasonable distinction in content. Hence, openness incorporates both openness and intellect as particular aspects. Given the selection of items of the applied BFI-K, there seems to be a bias towards the aspect of openness as only one of five items target intellect. In contrast, comprehensive questionnaires assessing domains and facets like NEO-PI-R and NEO-FFI [35] as applied in the discussed studies cope with both aspects. We conclude that we were not able to obtain strong intercorrelations of latent variables with openness, as we did not assess the scope of this dimension. It has to be noted that the correlations of BFI-K scales with those of the full Big Five Inventory (BFI) range from 0.85 (agreeableness) to 0.93 (neuroticism). While the internal consistencies of the BFI-K are naturally lower than those of the BFI, the retest reliabilities are quite comparable (0.80–0.93 across 6 weeks for the BFI vs. 0.76–0.93 for the BFI-K) [49]. Together with economic reasons, the BFI-K seemed highly appropriate. Furthermore, neither extraversion nor agreeableness were consistently associated with latent variables of our model of cognitive effort investment. This might not be surprising when considering the comparably low correlations, i.e. zero to almost moderate, with manifest variables previously reported by other researchers [14, 18, 24, 27, 36]. In summary, our model of cognitive effort investment allows for testing global as well as specific hypotheses in the context of effort investment.

## Limitations

Among the limitations of our study, sample composition may be the most crucial. As we aimed at examining dispositional differences of the general population including a wide age span and different educational backgrounds in Study 1, we used different recruitment approaches like social networks, online websites and advertisements. Nevertheless, the sample we complied was biased regarding age, sex, education. The same holds for Study 2, where we recruited at our university. Thus, the results presented here need to be replicated in the general population. In addition, the results of both studies are based on self-reported data and thus are subject to social desirability bias. Future studies should therefore strive to obtain peer-report data as well. Furthermore, the selection of traits as well as the hierarchical nature was based on pragmatic considerations. There may be additional relevant and informative variables that we did not include. For example, sensation seeking, daydreaming, cognitive processing capacity, willpower, or mindfulness meditation are supposed to affect the cost–benefit analysis on an individual basis [59]. A study of Malesza and Ostaszewski [60] revealed a negative correlation of effort discounting with harm avoidance and reward dependence, respectively, and a positive one with persistence. Nevertheless, the model presented here can be seen as a starting point for the construction of a more comprehensive model of cognitive effort investment whose hierarchical nature requires further specification.

One may argue that the use of only two indicators per latent variable is critical. While we agree in principle, we aimed at a model as simple as possible to be of practical use for research into individual differences in cognitive effort investment. We also considered principal components analysis over the items resulting in four indicators per primary factor. Here, the model fitted good, but worse than our simple model. The comparison of this model with the one without the second-order factor arrived at the same results, i.e., no significant difference in fit. As this approach is sample specific and, therefore, hardly transferable, we preferred the simpler model. In our future research, we will consider finding short scales that assess cognitive motivation and self-control in order to supplement our model in a way that retains the simplicity and practicality of the model, but at the same time is statistically sounder.

## Significance

Our work extends our current knowledge on traits related to effort investment and, thus, contributes to better understanding the role of individual differences in high demanding situations that require goal orientation. This new knowledge might improve predictions of individual control adjustments across a variety of tasks. As to the theoretical significance, the examined traits allow to discriminate individuals regarding their motivation to invest effort in specific tasks and, thus, their goal-directed behavior. Consequently, decision-making does not fully rely on cost–benefit analyses but individual factors have a significant influence. Although there are effort-discounting models and theories, these typically do not account for individual differences [7, 8], or only for particular ones, like cognitive skills [61]. Instead of treating interindividual variability as noise, we consider it useful to integrate dispositional individual differences in effort investment into present and yet to be developed motivational models to further improve our understanding about the underlying mechanisms of goal-directed behavior.

## Conclusions

With the present work, we expanded the comprehension of NFC (e.g. NFC shares both *seek* and *conquer* aspects) and showed that NFC and self-control share a common aspect of goal-directedness, although they seem distinct at first glance. Thus, both traits contribute to the understanding of individual differences in effort-related decisions. The established model of cognitive effort investment integrates traits that play a role in effort-related decision-making. This overarching construct allows the discrimination and prediction on cognitive motivation and effortful self-control. The construct relates to other important concepts in differential psychology as predicted. Building on this evidence, we plan to develop an economic instrument that accesses cognitive effort investment. Our model is an excellent starting point for more systematic inquiries into the role of dispositional cognitive effort investment in the modulation of effort-avoidant behaviors. So far, we could not detect any replication report of the seminal findings by Westbrook et al. [11] and Kool et al. [12]. We consider relating dispositional differences in cognitive effort investment to actual effort investment in task processing using objective effort indicators as an important endeavor. Successful replications of such a relationship would open an exciting avenue for further research, i.e., the integration of traits related to cognitive effort investment into cost–benefit-models like the opportunity-cost model [7], the expected value of control model [8] or other recent computational accounts like the active inference framework [62] in order to predict individual goal-directed behavior.

## Supplementary information

**Additional file 1:** Supplement A: *Sociodemographic Details (Study 1 & 2).* Supplement B: *Construct Validity of the Questionnaires Relevant for the Construct of Cognitive Effort Investment.* Supplement C:*Results of the Shapiro–Wilk Normality Test and Descriptive Statistics of All Variables (Study 1, N* = *613).* Supplement D:*Additional Packages Used With R for Statistical Analyses.* Supplement E:*Results of Shapiro–Wilk Normality Test and Descriptive Statistics of All Variables (Study 2, N = 244). *

## Data Availability

The datasets generated and/or analyzed during Study 1 and 2 study as well all analysis scripts are available in the OSF repository (https://osf.io/ujd36).

## Abbreviations

ATQ: Adult Temperament Questionnaire
BFI: Big Five Inventory
BFI-K: Big Five Inventory Short Form
CFA: Confirmatory factor analysis
CFI: Comparative Fit Index
COM: Cognitive motivation (referred on in figure only)
ECO: Effortful control (referred on in figure only)
ESC: Effortful self-control (referred on in figure only)
FFM: Five factor model of personality
INT: Intellect (referred on in figure only)
NFC: Need for cognition
RMSEA: Root mean square error of approximation
SCS: Self-control (referred on in figure only)
SRMR: Standardized root mean square residual
TU: Technische Universität
