# Supplementary material for: Dispositional individual differences in cognitive effort investment: establishing the core construct
Source: BMC Psychol. 2021 Jan 22;9:10. doi: 10.1186/s40359-021-00512-x (PMC7821547; doi:10.1186/s40359-021-00512-x)
Supplement: Supplementary file 1 — Additional file 1: Supplement A: Sociodemographic Details (Study 1 & 2). Supplement B: Construct Validity of the Questionnaires Relevant for the Construct of Cognitive Effort Investment. Supplement C:Results of the Shapiro–Wilk Normality Test and Descriptive Statistics of All Variables (Study 1, N = 613). Supplement D:Additional Packages Used With R for Statistical Analyses. Supplement E:Results of Shapiro–Wilk Normality Test and Descriptive Statistics of All Variables (Study 2, N = 244). [file 40359_2021_512_MOESM1_ESM.docx]

**Dispositional Individual Differences in Cognitive Effort Investment: Establishing the Core Construct**

Supplemental Materials

**Corinna Kührt*, Sebastian Pannasch, Stefan J. Kiebel, Alexander Strobel**

Faculty of Psychology, Technische Universität Dresden

**Supplement A**

Sociodemographic Details (Study 1 & 2)

Table A1

Age distribution (Study 1, *N* = 599; Study 2, *N* = 244)

| Study | Mean | SD | Median | Trimmed | Mad | Min | Max | Range | Skew | Kurtosis | SE |
| --- | --- | --- | --- | --- | --- | --- | --- | --- | --- | --- | --- |
| 1 | 29.12 | 10.83 | 26 | 27.21 | 5.93 | 14 | 92 | 78 | 1.75 | 3.34 | 0.44 |
| 2 | 23.39 | 4.28 | 22 | 22.79 | 2.97 | 18 | 39 | 21 | 1.34 | 1.71 | 0.27 |

Table A2

Sex distribution (Study 1 & 2) as absolute and percentage values

|  | Study 1 | |  | Study 2 | |
| --- | --- | --- | --- | --- | --- |
|  | Absolute | Percentage |  | Absolute | Percentage |
| Female | 430 | 70.15 |  | 175 | 71.72 |
| Male | 180 | 29.36 |  | 69 | 28.28 |
| NA’s | 3 | 0.49 |  | 0 | 0 |
| Total | 613 | 100 |  | 244 | 100 |

Table A3

Career statement (Study 1 & 2) as absolute and percentage values

|  | Study 1 | |  | Study 2 | |
| --- | --- | --- | --- | --- | --- |
| Characteristic | Absolute | Percentage |  | Absolute | Percentage |
| Graduation |  |  |  |  |  |
| Certificate of Secondary Education | 12 | 1.96 |  | 0 | 0 |
| General Certificate of Secondary Education | 73 | 11.91 |  | 2 | 0.82 |
| Matriculation Standard | 519 | 84.67 |  | 242 | 99.18 |
| Other | 9 | 1.47 |  | 0 | 0 |
| Total | 613 | 100 |  | 244 | 100 |
| Qualification |  |  |  |  |  |
| Other | 7 | 1.14 |  | Not assessed | |
| No job qualification | 34 | 5.55 |  |  |  |
| Apprentice | 11 | 1.79 |  |  |  |
| Student | 195 | 31.81 |  |  |  |
| Professional training qualification/ apprenticeship | 105 | 17.13 |  |  |  |
| Higher training qualification | 24 | 3.92 |  |  |  |
| Graduate degree | 237 | 38.66 |  |  |  |
| Total | 613 | 100 |  |  |  |
| Field of Study |  |  |  |  |  |
| Business, economics and law | 72 | 15.89 |  | Not assessed | |
| Civil and environmental engineering | 44 | 9.71 |  |  |  |
| Engineering sciences | 35 | 7.73 |  |  |  |
| Humanities and social sciences | 105 | 23.18 |  |  |  |
| Medicine and health | 23 | 5.08 |  |  |  |
| Other | 8 | 1.77 |  |  |  |
| Psychology | 124 | 27.37 |  |  |  |
| Science | 24 | 5.30 |  |  |  |
| Teachers training | 18 | 3.97 |  |  |  |
| Total | 453 | 100 |  |  |  |
| NA‘s | 160 |  |  |  |  |
| Current Job |  |  |  |  |  |
| No job | Not assessed | |  | 3 | 1.23 |
| Apprenticeship |  |  |  | 2 | 0.82 |
| Student |  |  |  | 209 | 85.66 |
| Employee |  |  |  | 26 | 10.66 |
| Freelancer |  |  |  | 4 | 1.64 |
| Total |  |  |  | 244 | 100 |

**Supplement B**

Construct Validity of the Questionnaires Relevant for the Construct of Cognitive Effort Investment

*NFC*. Correlations of NFC with other dispositional differences demonstrate good construct validity [1]. For instance, NFC correlates negatively with dogmatism (-.23 < *r* < -.24), simplification (*r* = -.26) and need for closure (*r* = -.26); it reveals positive relations to objectivism (*r = .47)*, intrinsic motivation (*r* = .57) and openness to ideas (*r* = .78). The applied short version of the German NFC Scale shows a positive correlation with scientific interest (*r* = .55) and achievement motivation (*r* = .57), but a negative one to failure motivation (*r* = -.20) [2].

*Intellect*. Mussel [3] demonstrated construct validity of the Intellect Scale in relation to the International Personality Item Pool: intellect correlates highly with intellect/imagination (*r* = .78) and moderately with conscientiousness (*r* = .37). It reveals either small or no relations to extraversion (*r* = .12), emotional stability (*r* = .21) and agreeableness (*r* = .01).

*Self-control*. The Brief Self-Control Scale reveals weak associations with perfectionism (-.29 < *r* < .20), extraversion (*r* = .09) and openness to experience (*r* = .11) and correlates positively with conscientiousness (*r* = .48), emotional stability (*r* = .42) and agreeableness (*r* = .29) [4]. The SCS-K-D correlates positively with self-regulation (*r* = .48) and self-esteem (*r* = .29), but negatively with perfectionism (*r* = -.28), procrastination (*r* = -.62), expression of aggression (*r* = -.35) [5].

*Effortful Control*. Wiltink et al. [6] demonstrated construct validity of the ATQ basing on a consistent pattern of relations to dispositions and interpersonal problems. As to effortful control, it reveals a negative correlation with neuroticism (*r* = -.55), no one with openness (*r* = .00) positive ones with conscientiousness (*r* = .58), agreeableness (*r* = .30) and extraversion (*r* = .27).

**Supplement C**

Additional Packages Used With R for Statistical Analyses

| Package | Version | Reference |
| --- | --- | --- |
| pwr | 1.2.2 | Champely S, Ekstrom C, Dalgaard P, Gill J, Weibelzahl S, Anandkumar A, . . . De Rosario H. pwr: Basic functions for power analysis. Power analysis functions along the lines of Cohen (1988); 2018. https://github.com/heliosdrm/pwr. |
| psych | 1.8.12 | Revelle W. psych: Procedures for psychological, psychometric, and personality research. Evanstone, Illinois; 2018. https://CRAN.R-project.org/package=psych. |
| lavaan | 0.6.5 | Rosseel Y. Lavaan: An R package for structural equation modeling. J Stat Softw 2012;48(2):1-36. |
| semTools | 0.5.2 | Jorgensen TD, Pornprasertmanit S, Schoemann AM, Rosseel Y. semTools: Useful tools for structural equation modeling. R package version 0.5-2; 2019. https://CRAN.R-project.org/package=semTools. |
| shape | 1.4.4 | Soetaert K. shape: Functions for Plotting Graphical Shapes, Colors; 2018. https://CRAN.R-project.org/package=shape. |
| papaja | 0.1.0.9842 | Aust F, Barth M, Diedenhofen B, Stahl C: papaja: Prepare reproducible APA journal articles with R Markdown; 2019. https://github.com/crsh/papaja. |

**Supplement D**

Results of the Shapiro-Wilk Normality Test and Descriptive Statistics of All Variables (Study 1, *N* = 613)

|  |  | Raw | |  | Normalized | | | | | | | |
| --- | --- | --- | --- | --- | --- | --- | --- | --- | --- | --- | --- | --- |
|  |  | Shapiro-Wilk Test | |  | Shapiro-Wilk Test | |  |  |  |  |  |  |
| Variable | | W | p |  | W | p | Mean | SD | Min | Max | Skew | Kurtosis |
| 1 | NFC | 0.992 | 0.003 |  | 0.999 | 0.996 | 0 | 0.997 | -3.085 | 3.085 | -0.001 | -0.086 |
| 2 | Intellect | 0.988 | 0 |  | 0.999 | 0.958 | -0.001 | 0.996 | -3.085 | 2.629 | -0.013 | -0.119 |
| 3 | Seek | 0.981 | 0 |  | 0.997 | 0.2 | -0.002 | 0.992 | -3.085 | 2.297 | -0.039 | -0.183 |
| 4 | Conquer | 0.989 | 0 |  | 0.998 | 0.671 | -0.001 | 0.994 | -3.085 | 2.517 | -0.019 | -0.139 |
| 5 | Self-Control | 0.996 | 0.169 |  | 0.998 | 0.834 | 0 | 0.996 | -3.085 | 3.085 | 0.001 | -0.087 |
| 6 | Effortful Control | 0.997 | 0.219 |  | 0.999 | 0.999 | 0 | 0.997 | -3.085 | 3.085 | 0 | -0.08 |
| 7 | General Self-Efficacy | 0.979 | 0 |  | 0.995 | 0.027 | -0.001 | 0.991 | -2.788 | 2.517 | -0.015 | -0.146 |
| 8 | Neuroticism | 0.978 | 0 |  | 0.989 | 0 | -0.002 | 0.981 | -2.517 | 2.132 | -0.038 | -0.289 |
| 9 | Extraversion | 0.975 | 0 |  | 0.99 | 0 | -0.002 | 0.983 | -2.7 | 2.297 | -0.033 | -0.241 |
| 10 | Openness | 0.953 | 0 |  | 0.977 | 0 | -0.01 | 0.966 | -3.085 | 1.653 | -0.161 | -0.385 |
| 11 | Agreeableness | 0.983 | 0 |  | 0.989 | 0 | 0.001 | 0.983 | -2.297 | 3.085 | 0.021 | -0.216 |
| 12 | Conscientiousness | 0.976 | 0 |  | 0.985 | 0 | -0.003 | 0.981 | -2.905 | 2.219 | -0.049 | -0.19 |

**Supplement E**

Results of Shapiro-Wilk Normality Test and Descriptive Statistics of All Variables (Study 2, *N* = 244)

|  |  | Raw | |  | Normalized | | | | | | | |
| --- | --- | --- | --- | --- | --- | --- | --- | --- | --- | --- | --- | --- |
|  |  | Shapiro-Wilk Test | |  | Shapiro-Wilk Test | |  |  |  |  |  |  |
| Variable | | W | p |  | W | p | Mean | SD | Min | Max | Skew | Kurtosis |
| 1 | NFC | 0.988 | 0.036 |  | 0.999 | 1 | 0 | 0.994 | -2.8 | 2.8 | -0.001 | -0.153 |
| 2 | Intellect | 0.991 | 0.12 |  | 0.999 | 1 | 0 | 0.994 | -2.8 | 2.8 | 0.001 | -0.164 |
| 3 | Seek | 0.988 | 0.035 |  | 0.997 | 0.968 | 0 | 0.992 | -2.604 | 2.8 | 0.005 | -0.181 |
| 4 | Conquer | 0.989 | 0.072 |  | 0.998 | 0.997 | 0 | 0.993 | -2.8 | 2.8 | 0 | -0.16 |
| 5 | Self-Control | 0.991 | 0.135 |  | 0.997 | 0.914 | 0 | 0.991 | -2.8 | 2.604 | -0.003 | -0.18 |
| 6 | Effortful Control | 0.995 | 0.637 |  | 0.999 | 1 | 0 | 0.994 | -2.8 | 2.8 | 0.001 | -0.159 |
| 7 | General Self-Efficacy | 0.99 | 0.112 |  | 0.993 | 0.307 | 0 | 0.988 | -2.8 | 2.8 | -0.001 | -0.147 |
| 8 | Neuroticism | 0.984 | 0.007 |  | 0.99 | 0.095 | -0.001 | 0.984 | -2.8 | 2.475 | -0.013 | -0.212 |
| 9 | Extraversion | 0.969 | 0 |  | 0.983 | 0.005 | -0.005 | 0.973 | -2.475 | 1.893 | -0.079 | -0.376 |
| 10 | Openness | 0.957 | 0 |  | 0.977 | 0 | -0.009 | 0.965 | -2.8 | 1.669 | -0.147 | -0.413 |
| 11 | Agreeableness | 0.981 | 0.003 |  | 0.989 | 0.071 | 0 | 0.983 | -2.604 | 2.8 | -0.005 | -0.195 |
| 12 | Conscientiousness | 0.976 | 0 |  | 0.981 | 0.002 | -0.002 | 0.975 | -2.604 | 2.123 | -0.043 | -0.262 |

**References**

1. Cacioppo JT, Petty, R. E.; Feinstein, J. A.; Jarvis, W. B. G. Dispositional Differences in Cogntive Motivation: The Life and Times of Individuals Varying in Need for Cognition. Psychol Bull. 1996;119(2):197-253.
2. Bless H, Wänke M, Bohner G, Fellhauer RL, Schwarz N. Need for Cognition: Eine Skala zur Erfassung von Engagement und Freude bei Denkaufgaben [need for cognition: A scale measuring engagement and happiness in cognitive tasks]. Soc Psychol (Gott) 1994;25:147-154.
3. Mussel P. Intellect: A theoretical framework for personality traits related to intellectual achievements. J Pers Soc Psychol 2013;104(5):885-906. doi:10.1037/a0031918.
4. Tangney JP, Baumeister RF, Boone AL. High self-control predicts good adjustment, less pathology, better grades, and interpersonal success. J Pers 2004;72(2):271-324.
5. Bertrams A, Dickhäuser O. Messung dispositioneller Selbstkontroll-Kapazität. Diagnostica 2009;55(1):2-10. doi:10.1026/0012-1924.55.1.2.
6. Wiltink J, Vogelsang U, Beutel ME. Temperament and personality: The German version of the Adult Temperament Questionnaire (ATQ). Psychosoc Med 2006;3:1-13. http://www.egms.de/en/journals/psm/2006-3/psm000030.shtml.
